# Traumatic, Irideremia, and Aphakia

**Published:** 1870-09

**Authors:** C. Hixson

**Affiliations:** Formerly Prof. of Ophthamology in the Kansas City College of Physicians and Surgeons


ARTICLE XXIX.

### TRAUMATIC, IRIDEREMIA, AND APHAKIA.

By C. HIXSON, M.D., formerly Prof. of Ophthamology in the Kansas City College of Physicians and Surgeons.

The infrequency of cases like the following is a sufficient apology for its publication. Mr. B., of Kansas, is a sawyer by occupation, and sometime in January last, was struck by a small chip hurled with great velocity from the saw, which entered the centre of the right eye, making an incision of more than three-fourths of the diameter of the cornea. Being remote from any professional assistance, his wife did the surgery in the case. Cold applications were made at intervals for several days, when what he describes as strings of pus was found in the wound, and which his wife pulled away with considerable pain on the part of the patient. The eye gradually improved, and to-day I find the following interesting condition of affairs: There is, extending in a vertical line, embracing about three-fourths of the diameter of the cornea, a firm, opaque cicatrix. There is not a vestige of the iris to be seen in the injured eye. The iris of the left eye is a very light grey, and contrasted with the deep, dark, blackness of the injured eye, is remarkable. The only thing seen by lateral illumination or by the Ophthalmoscope is a few opaque filaments of the capsule. There is complete aphakia. Without a glass, the patient has only quantative perception of light—everything is blurred and indistinct. With a cataract glass and through a stenopaic hole, he can read No. 4 Sneller at 18 inches. He is very much annoyed by the inability to focus the rays of light equally in the two eyes, and I hope by a stenopaic catacact glass to be able to do away with this difficultuy to a considerable extent, if not entirely.

